# Monatomic ions influence substrate permeation across bacterial microcompartment shells

**DOI:** 10.1038/s41598-023-42688-9

**Published:** 2023-09-21

**Authors:** Daniel S. Trettel, Chris Neale, Mingfei Zhao, S. Gnanakaran, C. Raul Gonzalez-Esquer

**Affiliations:** 1 Biosciences Division, Microbial and Biome Sciences Group, Los Alamos National Laboratory, Los Alamos, NM USA; 2 Theoretical Division, Theoretical Biology and Biophysics Group, Los Alamos National Laboratory, Los Alamos, NM USA

**Keywords:** Molecular modelling, Biophysical chemistry

## Abstract

Bacterial microcompartments (BMCs) are protein organelles consisting of an inner enzymatic core encased within a selectively permeable shell. BMC shells are modular, tractable architectures that can be repurposed with new interior enzymes for biomanufacturing purposes. The permeability of BMC shells is function-specific and regulated by biophysical properties of the shell subunits, especially its pores. We hypothesized that ions may interact with pore residues in a manner that influences the substrate permeation process. In vitro activity comparisons between native and broken BMCs demonstrated that increasing NaCl negatively affects permeation rates. Molecular dynamics simulations of the dominant shell protein (BMC-H) revealed that chloride ions preferentially occupy the positive pore, hindering substrate permeation, while sodium cations remain excluded. Overall, these results demonstrate that shell properties influence ion permeability and leverages the integration of experimental and computational techniques to improve our understanding of BMC shells towards their repurposing for biotechnological applications.

## Introduction

Bacterial microcompartments (BMCs) are protein based metabolic organelles present in a wide variety of bacterial phyla^1^. Typically 40–300 nm in diameter, all BMCs contain inner enzymatic cargo encased and protected within an outer protein shell^2^. BMCs are classified based on the internal chemistry they accommodate within their enzymatic core which can range from carbon fixation (carboxysomes) to the assimilation of niche metabolites like 1,2-propanediol or ethanolamine (metabolosomes)^1^. Despite their functional plasticity, functional BMCs rely on conserved structure and assembly principles.

BMC shells are icosahedral structures assembled from hexameric (BMC-H), trimeric (BMC-T) and pentameric (BMC-P) protein subunits^1,3^. These subunits all present as relatively flat tiles with characteristic concave (outwards) and convex (inwards) faces^4^. BMC-H and BMC-T are thought to tesselate into curved sheets to form the facets of the icosahedral BMC while BMC-P cap the vertices^5,6^. Once assembled, substrates and cofactors selectively permeate through the shell predominately at pores within the BMC-H and, potentially, BMC-T structures^7–11^. The natural propensity of BMC shells to self-assemble and act as selective barriers have garnered considerable interest for their potential to create designer BMCs with tailored enzymatic core function for their use in biomanufacturing or to enhance carbon fixation^12–14^.

A nuanced understanding of the BMC pores must be developed to usher in the next generation of BMC biotechnological applications. The shell pores have been implicated in selectively enabling influx of metabolites and sequestering toxic internal intermediates^7,15^. For example, the propanediol utilization (Pdu) BMC shell allows passage of 1,2-propanediol which is then converted to propanal, a reactive intermediate, that is retained by the shell^7,16^. Other BMC pores, like in carboxysomes, also inhibit influx of O_2_ which would otherwise lead to wasteful photorespiration byproducts^17^. Several studies have described the permeation of substrates across BMC-H pores using experimental^7,17^ and molecular dynamics (MD) simulations^18–20^. The pores of shell proteins usually present a highly charged local environment about the central pore, such as in the case of PduA and CcmK1, which both have strong basic character^20,21^. Accordingly, the charge of substrates and pH^22^ are known to be critical parameters in permeation but the potential importance of ions in these events is poorly understood.

Analogously, we hypothesized that ions interact with BMC pores according to their charge and thus can affect substrate permeation. Herein, we analyzed the effect of NaCl on substrate permeation rates by quantifying the propanediol dehydratase (PduCDE) activity rate in intact versus broken Pdu BMC shells in vitro. Higher NaCl concentrations were found to negatively affect S-1,2-propanediol (hereafter 1,2-propanediol) permeation across the shell. Molecular dynamics simulations of the BMC-H shell protein PduA (a main component in the Pdu BMC) provided mechanistic insights on the influence of NaCl on substrate-pore interactions. Cl^-^ ions were found to be enriched at the pore and reduce 1,2-propanediol permeation rates relative to simulations containing no NaCl, slowing 1,2-propanediol permeation. These results bring new insight into the behavior of BMC pores with implications in how we understand, design, and apply BMC biotechnology for novel applications in the future.

## Results

### Propanediol dehydratase activity within Pdu compartments serves as a proxy for shell permeability

**Figure 1:**
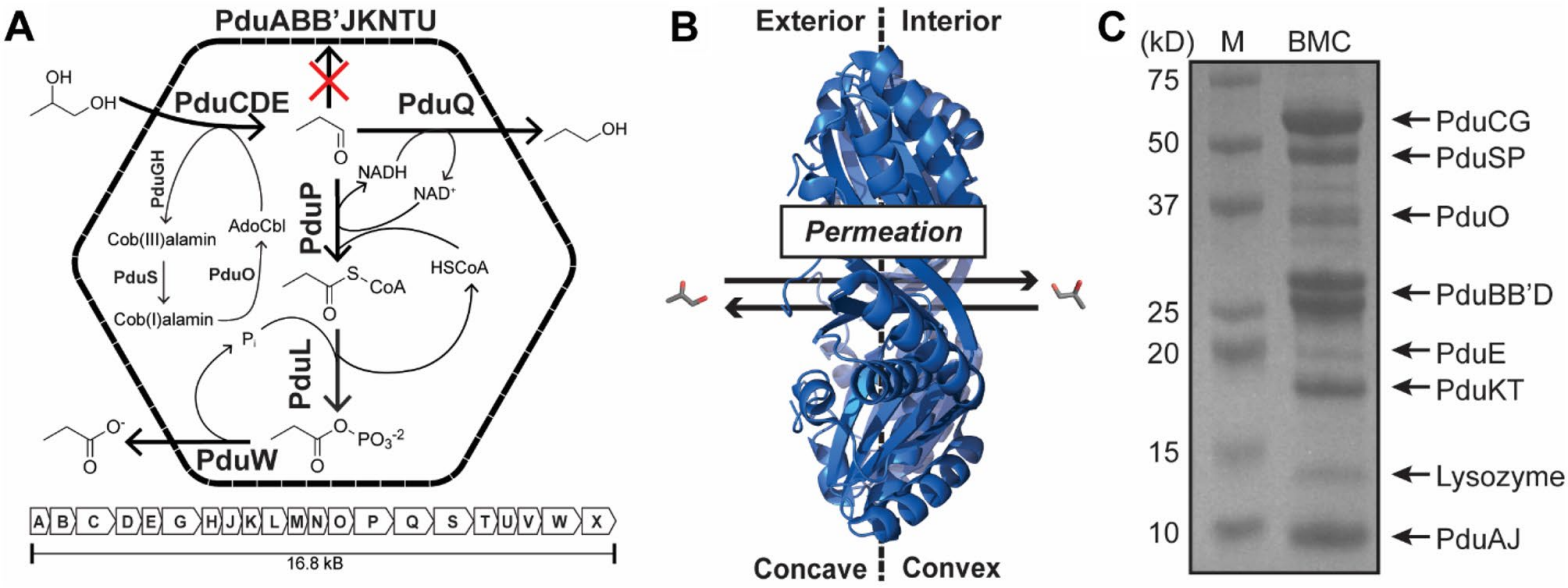
The model 1,2-propanediol utilization (Pdu) BMC from Salmonella enterica used in this study. (**A**) A schematic of the Pdu BMC system. It is composed of at least 8 distinct shell proteins that are thought to mediate 1,2-propanediol influx and inhibit propanal efflux (red X). A representation of the operon structure is also shown. (**B**) All shell proteins, including PduA shown (PDB: 3NGK), have a concave and convex surface corresponding to the outside and inside of the BMC, respectively. We consider permeation as any movement of a permeant from one side to the other via the pore. (**C**) An SDS-PAGE profile of S. enterica Pdu BMCs purified from a heterologous E. coli host used for this study. The full uncropped or edited gel can be found in Fig. S1. Major protein components are identified.

We chose the Pdu BMC from *Salmonella enterica*^23^ as our model system for probing shell permeability (Fig. 1A). In this system, the shell (consisting of the proteins PduABB’JKNTU) allows the diffusion of 1,2-propanediol to the BMC lumen (Fig. 1B), which then gets converted to propanal by the signature enzyme propanediol dehydratase (PduCDE; EC 4.2.1.28) in a B_12_-dependent manner (Fig. 1A)^24^. Propanal, a reactive and damaging intermediate, is thought to be retained by the shell^25^ before conversion to propanol or propionyl-CoA by an alcohol or aldehyde dehydrogenase, respectively. Measurements of propanediol dehydratase activity have been previously used as an indirect proxy for shell permeation^7,16^. In this work, purified Pdu BMCs supplemented with 1,2-propanediol and B_12_ convert the substrate to propanal. Conveniently, propanal itself exhibits strong absorbance at 280 nm^26^ and is agnostic to NaCl content (Fig. S2) enabling spectrophotometric time-resolved detection. For this purpose, *S. enterica* Pdu BMCs were expressed in *E. coli*^23^ and successfully purified by differential centrifugation (Fig. 1C).

### Shell integrity is correlated with BMC function under various NaCl concentrations

**Figure 2:**
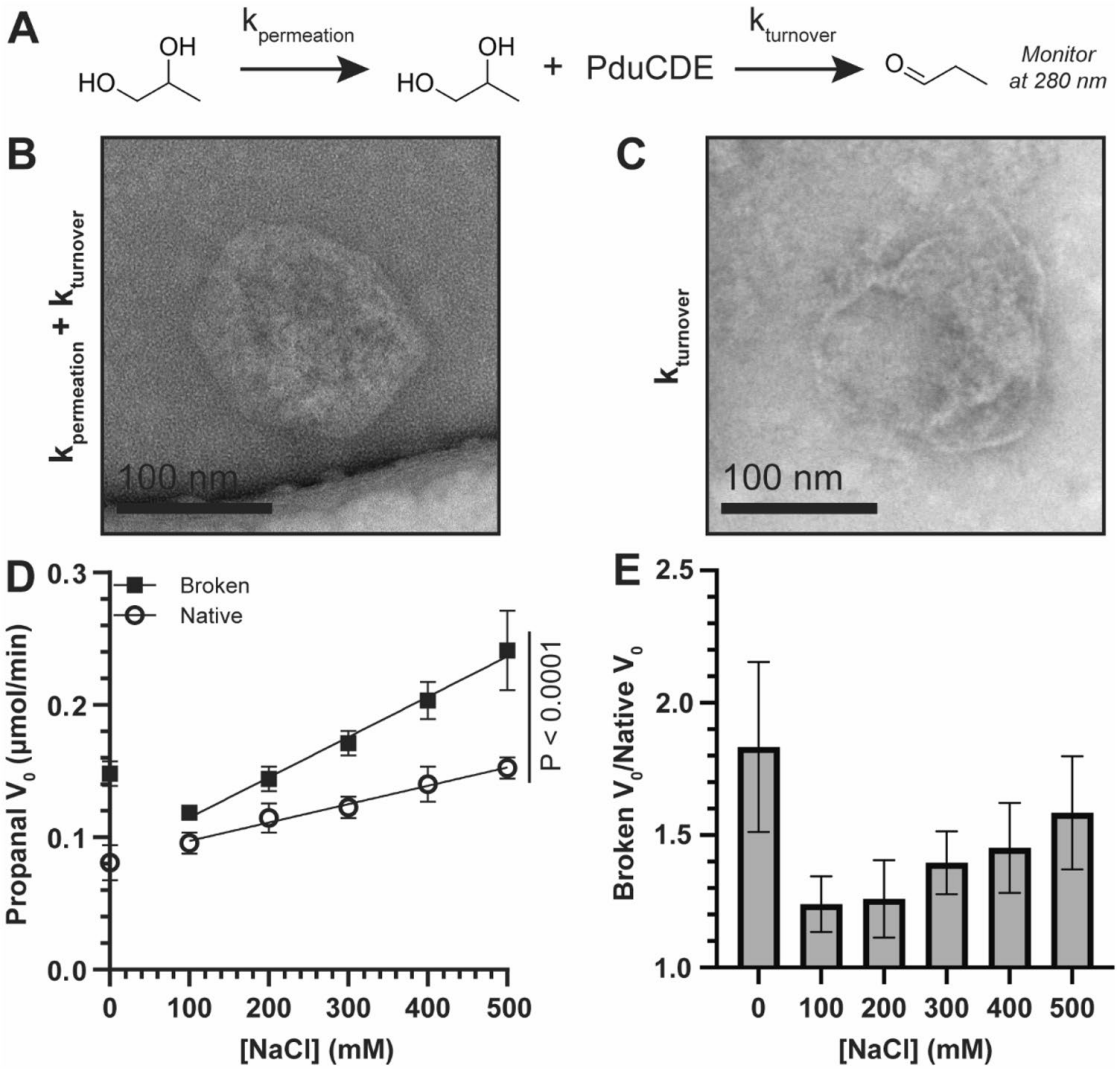
Salt influences the permeation of substrate across the Pdu BMC shell. (**A**) The apparent formation rate of propanal is dependent on the rate of permeation (k_permeation_) of 1,2-propanediol across the shell and the rate of catalysis (k_turnover_) by the signature complex PduCDE. Propanal formation is monitored at 280 nm. (**B**) Electron micrographs of purified, intact Pdu BMCs and (**C**) BMCs broken by sonication. Activities in intact BMCs are affected by both k_permeation_ and k_turnover_ while broken BMCs have k_permeation_ minimized leaving k_turnover_ as the main consideration. (**D**) The initial velocity (V_0_) of propanal formation was monitored as a function of [NaCl]. A linear regression was performed on points between 100–500 mM NaCl, leading to significantly different slopes (*P* < 0.0001). Assays were performed in quadruplicate. (**E**) The ratio of V_0_’s between broken and native samples are not constant as a function of [NaCl], indicating both that broken samples are faster and that k_permeation_ is negatively affected by increasing NaCl. Error in (**E**) was compounded from (**D**). Panels (**D**) and (**E**) were generated in GraphPad Prism 9.

The rate of propanal formation is influenced by the rate of 1,2-propanediol diffusion through the shell (k_permeation_) and the rate of enzymatic turnover (k_turnover_) by PduCDE (Fig. 2A). These conditions leave k_permeation_ inextricably tied to k_turnover_ and difficult to decouple in native BMCs. Therefore, comparing the apparent propanal formation rates between native and broken Pdu BMCs as a function of [NaCl] would allow us to better parse out the influence on k_permeation_ alone. To do so, a sample of Pdu BMCs was purified and split into two equivalent fractions with one being left unprocessed (Fig. 2B) and the other treated with brief sonication to disrupt the shell architecture (Fig. 2C)^7,27^. Intact BMCs exhibited typical polyhedral morphology, and thus the observed propanal formation rates are governed by both k_permeation_ and k_turnover_ (Fig. 2B). Broken BMCs, in contrast, show reduced electron density with less structured shell boundary (Fig. 2C). As such, the k_permeation_ term is minimized relative to native samples and the observed rates of propanal formation more closely reflect the activity of PduCDE alone (k_turnover_). Given this theoretical framework, differences in activity rate as a function of [NaCl] between intact and broken Pdu BMCs can be primarily attributed to how well the 1,2-propanediol substrate permeates the shell.

Native and broken Pdu BMCs were reconstituted in buffer containing coenzyme B_12_ and between 0 and 500 mM NaCl as appropriate. To measure activity rates, 1,2-propanediol was added to the reaction mix to initiate the reactions and propanal formation was monitored continuously at 280 nm for an hour (Fig. S3). All reaction conditions produced propanal except for a control that lacked B_12_ (Fig. S3). The initial velocities of propanal formation (V_0_) were calculated and plotted as a function of NaCl concentration (Fig. 2D). Native samples showed a linear increase in activity rate with increasing NaCl. The assay was run in parallel with Pdu BMCs broken by sonication to serve as a control for the influence of the shell layer. All sonicated samples were enzymatically active which suggests the shell breaking protocol does not significantly hinder enzyme stability. The activity rate of broken BMCs also showed an increase with NaCl and was always higher than intact samples. This latter observation is supported by the notion that the shell acts as a diffusive barrier, slowing permeation which in turn slows turnover. Broken BMC samples also showed decreasing reaction half-lives with increasing NaCl while intact samples were similar across all conditions (Fig. S3C). This agrees with a broken shell morphology; the observed higher turnover rates could result in quicker break down coenzyme B_12_^27,28^, limiting its availability, as components for its regeneration were not added to the reaction mix. Similarly, there is an increased potential for inactivation of diol dehydratase due to higher exposure to molecular oxygen^29,30^.

While both broken and intact samples exhibited increasing initial velocities (V_0_) with increasing NaCl, their trendlines differed significantly (*P* < 0.0001; Fig. 2D). The rate of activity gains as a function of NaCl were lower for the native samples relative to the broken. We expressed this disparity as the ratio of broken V_0_ to native V_0_ which increases with salt as opposed to staying constant (Fig. 2E). These data show that NaCl may act, through an unknown mechanism, as an enhancer for k_turnover_ since this is the only term governing the activity rate of broken BMCs. This rate increase is suppressed in native samples, which experience both k_turnover_ and k_permeation_ governed by an intact shell. Since k_turnover_ is presumably the same in both samples (as diol dehydratase exist inside and outside of a BMC context)^40^ then k_permeation_ is suggested to be decreasing with additional salt. Accordingly, the results may support the notion that NaCl negative effects the permeation of 1,2-propanediol.

### Molecular dynamics reveal that chloride ions modulate 1,2-propanediol permeation but not vice versa

**Figure 3:**
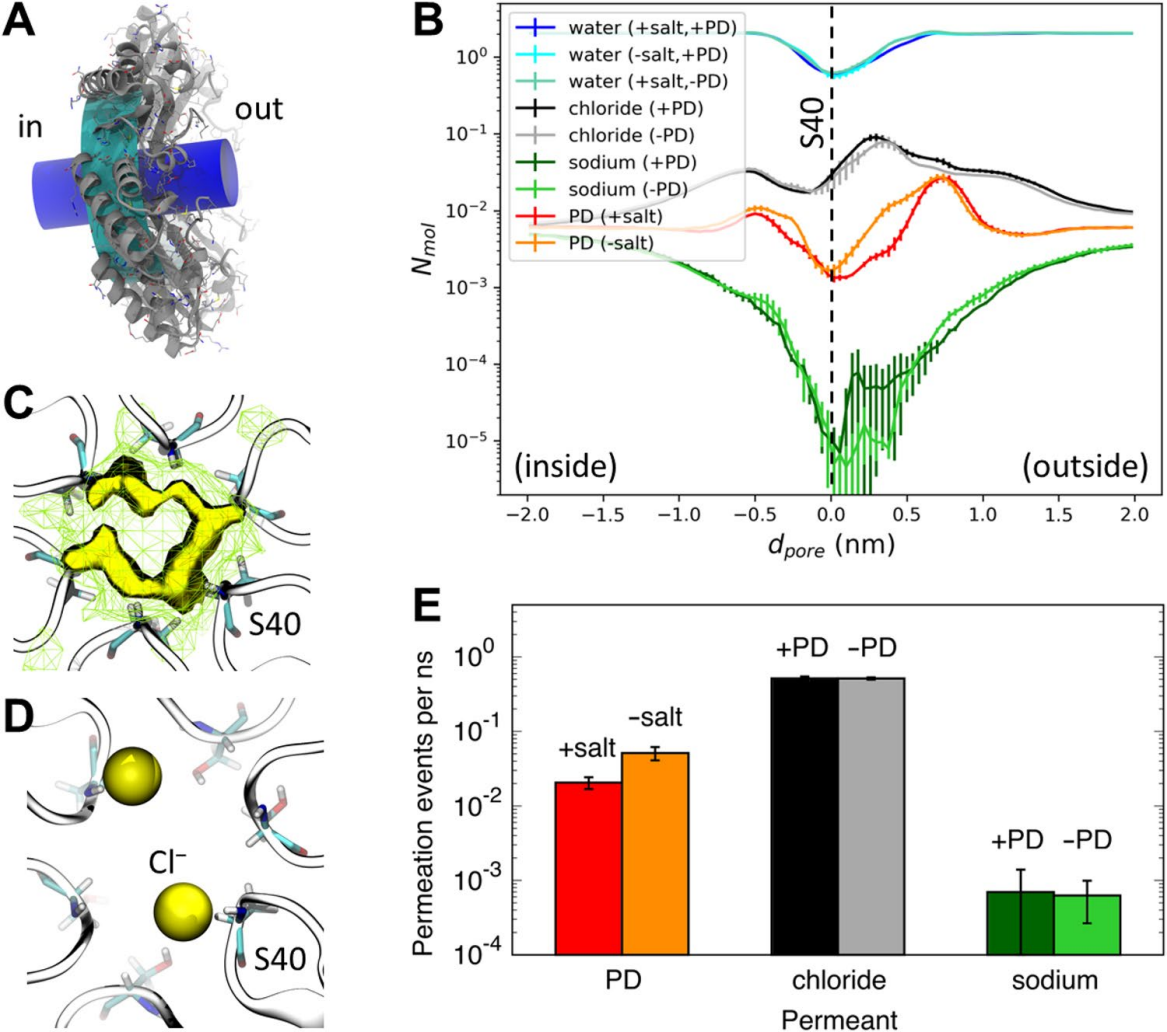
Molecular occupancy and permeation through WT PduA in MD simulations. (**A**) Graphical depiction of the geometrical definitions used to estimate (blue cylinder) *N*_*mol*_ and *d*_*pore*_ and (cyan cylinder) molecular permeation rates (see “Methods”). (**B**) Histograms of the average number of each molecular, *N*_*mol*_, at various displacements along a 0.7 nm-radius cylinder aligned with the PduA pore axis, *d*_*pore*_, referenced such that the average position of PduA S40 C_α_ atoms are at *d*_*pore*_ = 0 nm. Data shown separately for water and 1,2-propanediol (PD) in simulations with and without salt, and for Cl^-^ and Na^+^ in simulations with and without PD. Vertical bars represent standard errors across three repeat simulations. (**C**) Spatial distribution functions of Cl- in the WT PduA pore. Residue S40 is shown for clarity. Yellow grid lines and solid surface enclose regions with time- and ensemble-average Cl^-^ densities greater than 10- and 30-times bulk values, respectively. Data are from the three simulations lacking metabolites. (**D**) Representative conformation with two Cl^-^ ions in the pore, coordinated by backbone amide groups of S40 residues. (**E**) Rates of bidirectional molecular permeation through the PduA pore. Permeants are labeled on the abscissa and simulation conditions are noted above each bar.

Our in vitro experimental data suggest that salt may negatively influence substrate permeation. However, the results are complicated by the inherently indirect observations for the permeation process. We next turned to molecular dynamics (MD) simulations to provide deeper molecular insight into our experimental observations. MD simulations of isolated PduA hexamers, in the presence or absence of either 1,2-propanediol or NaCl, were performed to assess the permeation of 1,2-propanediol through the central PduA pore. In our study, permeation is regarded as any movement of a metabolite through the pore in either direction (Fig. 1B). PduA (Uniprot: P0A1C7) was selected for modeling as it, along with its homolog PduJ, make up nearly 60% of the Pdu BMC by mass^31^ and allow extrapolation of MD results from isolated hexamers toward higher-order BMC shells. Systems contain either 175 mM 1,2-propanediol with or without 150 mM salt, or 150 mM salt and no 1,2-propanediol (Table 1). The concentration of 1,2-propanediol was selected to be similar to, but slightly greater than, the salt concentration in an attempt to reduce protein perturbation and increase sampling sufficiency. Three replicas of each of these systems were simulated for 1.5 µs each. A representative 1 ns simulation snippet can be found in Supplemental Movie 1. The number of solute molecules (N_mol_), pore axis (d_pore_) and pore traversal rates were based on geometrical definitions within a cylinder of 0.7 nm radius along the normal to the pore as depicted in Fig. 3A.

**Table 1:** Simulation systems.

System	Protein	[S-1,2-propanediol] (mM)	[propionaldehyde] (mM)	[NaCl] (mM)	Duration (μs per simulation)	No. repeats
WT^a^	WT	0	0	150	~ 1.5	3
WT, + PD^b^	WT	175	0	150	~ 1.5	3
WT, + PD, − salt	WT	175	0	0	~ 1.5	3
WT, + ALD	WT	0	175	150	~ 1.5	3
S40A, + PD	S40A	175	0	150	~ 1.5	3
S40A, + ALD	S40A	0	175	150	~ 1.5	3

^a^The sequence marked as WT omits N-terminal M_1_QQ_3_ and C-terminal S_93_Q_94_ residues, which are missing in the selected crystal structure.

^b^PD, 1,2-propanediol; ALD, propanal; salt, NaCl.

Quantification of water of the native PduA pore reveal a hydrated ~ 1 nm long constriction near the central GSG hairpin motif (Fig. 3B, blue). Although the GSG motif is more flexible than nearby residues, local structural deviations from the crystallographic conformation are of similar magnitudes in the presence and absence of metabolic permeants (Fig. S4). Whereas Cl^-^ ions are enriched near the pore, especially near the concave face, the outer surface of an intact BMC (Fig. 3B black), Na^+^ ions are strongly excluded (~ 100-fold; Fig. 3B, green). Within the timescales of simulations considered in this study, the presence of NaCl did not induce statistically significant changes in global hexamer shape (Figs. S5, S6, and Table S1) The density profile of 1,2-propanediol contains regions of enrichment bounding a slight depletion near the central constriction (Fig. 3B, red/orange). These profiles also reveal that the presence of 150 mM NaCl influences the distribution of 1,2-propanediol along the pore axis, with salt depressing the population of 1,2-propanediol on either side of the pore, especially on its outside face where Cl^-^ is most strongly enriched (Fig. 3B). Although the presence of 1,2-propanediol also slightly affects the distribution of Cl^-^ ions along the pore axis, this effect is of opposite direction (i.e., 1,2-propanediol further enriches Cl^-^ at the pore’s external face; Fig. 3B). However, the profiles of Cl^-^ density along the pore normal with and without 1,2-propanediol are sufficiently similar that these results enable the conclusion that Cl^-^ can preferentially exclude 1,2-propanediol but not vice versa. We presume that this largely one-way causality emerges due to the ~ tenfold difference in local equilibrium ([Cl^-^] is ~ 10 × higher than [1,2-propanediol]) in the central constriction (Fig. 3B). Although Cl^-^ ions are localized across the WT pores full diameter, they preferentially enrich around the periphery (Fig. 3C) near the backbone amide groups of S40 residues (Fig. 3D).

Direct assessment of pore traversal in these simulations reveals that these rates follow the pattern Cl^-^ > 1,2-propanediol > Na^+^ (Fig. 3E), sharing the ranking of decreasing molecular density near the pore constriction (Fig. 3B). Importantly, while the presence of 1,2-propanediol does not significantly affect the permeation rates of Cl^-^ or Na^+^ through WT PduA, the presence of salt does influence the permeation rate of 1,2-propanediol, cutting it roughly in half from 0.05 ± 0.01 permeation events per ns without salt to 0.02 ± 0.004 permeation events per ns with 150 mM NaCl (Fig. 3E).

Additional MD simulations reveal that, although 1,2-propanediol and propanal have similar density profiles along the WT PduA pore axis (Fig. S7C), the permeation rate of propanal in the presence of salt (0.04 ± 0.01 permeations events per ns; Fig. S7F, WT ALD) is approximately two-times that of 1,2-propanediol (Fig. S7E, WT PD) in 150 mM NaCl. This is in apparent conflict with results from simulations of PduA hexamers by Park et al.^19^ where no excess salt was considered. Those simulations indicated that the free-energy barrier to permeation is approximately 1 kcal/mol (from replica exchange umbrella samplings) or 1.5 kcal/mol (from metadynamics) larger for propanal than for 1,2propanediol which corresponds to a local density ratio between 3 and 6, respectively^19^. Furthermore, a previously reported S40A mutation^7^ reduces the amount of Cl^-^ (Fig. S2B) and increases the amount of both metabolites in the pore (Fig. S7C) and increases metabolite permeation rates by a factor of four (1,2-propanediol; Fig. S7E) or three (propanal; Fig. S7F).

## Discussion

This study sought to explore the influence of NaCl on substrate permeation events across BMC pores. We find that Cl^-^ ions are enriched at the PduA pore constriction, reducing 1,2-propanediol movement. These findings result from a union of experimental data and MD simulations. Our in vitro observations suggested that salt may negatively influence substrate permeation, but inherently indirect observations of the permeation process precluded a firm conclusion. Direct assessment of pore traversal and occupancy using MD simulations, however, supported these initial findings. In both instances, salt was found to be an inhibitor of 1,2-propanediol permeation.

One caveat is that the experimental data showed a significant decrease in activity in broken BMC samples when taken from 0 to 100 mM NaCl that was not observed in the native samples (Fig. 2D). We believe this stems from Na^+^ acting as an inhibitor of PduCDE^32^. Our MD results show that Na^+^ is significantly hindered from traversing the PduA pore (Fig. 3B). Therefore, we expect no decrease in activity in native samples due to Na^+^, which was observed in our in vitro experiments (Fig. 2D). Further, our in vitro data showed salt acts as a general enhancer for the observed activity rates. While this effect is suppressed in native BMCs relative to broken BMCs, suggesting the intact shell governing k_permeation_ is responsible, we cannot firmly attribute a cause for this general rate enhancement. We speculate that while Na^+^ may be an inhibitor of PduCDE, salt overall may alter factors like the hydration state or organization of Pdu complexes in a positive manner.

Consistent with our observations of Na^+^ depletion at the PduA pore, simulations of carboxysome shell proteins by Faulkner et al*.* indicate that monatomic cations (K^+^, specifically) are preferentially excluded from the CcmK2 pore^18^. Moreover, simulations of carboxysome hexamers CsoS1A and CcmK4 by Mahinthichaichan et al*.* demonstrated Cl^-^ binding to backbone amides of central protomeric β-turns and preferential pore localization of the anion HCO_3_^−^^20^. Several aspects of the theoretically derived conclusions of Park et al*.* are consistent with our observations though the effect of salt was not considered in the former^19^. The profiles of potential of mean force of 1,2-propanediol traversal through the PduA hexamer pore from that study is consistent with the molecular density observed in our study. Both studies show that a depletion or an unfavored energetic contribution to observing the metabolite near the central constriction and an enrichment of metabolite of favorable energetic contribution on the outside of the shell (> 5 Å). We are not able to consider the effect of salt on relative permeabilities of 1,2-propanediol and propanal since simulations of Park et al*.* did not include excess salt^19^. Experimental evidence from Chowdhury et al. agrees with our findings that propanal can escape the WT Pdu BMC and that an S40A mutation in PduA hastens this escape (Fig. S7F)^7^. With our results, we reason that, given the similar chemical properties of propanediol and propanal, that propanal may be too rapidly turned over within the BMC lumen to ever be effectively selected against by the protein shell.

The ability of PduA and carboxysomal CsoS1A and CcmK4 to preferentially bind negatively charged species (HCO_3_^−^, Cl^−^)^20^ indicates this may be a widely distributed function of BMC-H proteins. Indeed, other BMC-H proteins, such as EutM from *Escherichia coli*^33^ and CcmK1 and CcmK2 from *Synechocystis* sp. PCC6803^9^, also coordinate sulfate groups at their pore when crystallized. Despite having slightly different pore-lining residues (S, T, G, or S40A tested herein [Figs. S7E, S7F]) they all share a highly positive concave surface near the pore itself. Further, our findings here strongly suggest that these anions, specifically Cl^-^, can modulate substrate permeation and therefore compartment activity. Whether any of these anions are specific targets of BMC-H pores, whether different BMCs would prefer different anions, and why/whether this method of modulation is biologically important are all open questions. The Pdu BMC may also coordinate PO_4_^3−^ to enable its permeation and use by the internal phosphotransacetylase PduL^34^. Similarly, this preference for anionic species may encourage export/general permeation of the end-products propionyl-phosphate or propionate from the Pdu BMC. In any case, it appears that BMC-H shell proteins may equally be tuned for selection of ions as much as they may be for specific metabolites and that these ions further attune the shell for metabolite passage. Accordingly, it is tempting to speculate that permeation itself is an environmentally responsive event.

We note that other areas of a BMC, beyond the pore studied here, may also be responsible for passage of permeants and impact our interpretations. For example, BMC-T^dp^ proteins can stack to form internal cavities which, along with the observation of their relatively large central pores, has invited speculation that they enable passage of larger cofactors^35–37^. Further, BMC structural dynamics, an emerging line of study, could allow for transient holes within the BMC shell^38,39^. These aspects, among others, of the BMC shell still have undefined roles in permeation. Our computational study may similarly be impacted by chosen force fields, lack of sampling, or by considering the permeability of PduA hexamer in isolation without the neighboring shell proteins that make up the BMC. Our in vitro model also depends on the notion that the signature complex behaves similarly between native and broken BMCs. However, these enzymes exist coupled and decoupled from BMC shells^40^, suggesting similar enzymatic capabilities within each context.

Our study suggests another level of consideration and complexity to studying and designing permeation events across BMC shells. Key to studying, designing, and implementing these considerations in the future are a marriage of experimental testing and MD simulations, like those used here. Development of MD simulations that assess the influence of specific mutations on permeation rates of desired metabolites and ions will be key to future work. This work should consider not just the pore constriction, but also the pore-adjacent regions in a structurally non-disruptive manner.

## Methods

### Pdu BMC Purification

*S. enterica* Pdu BMCs were purified from a heterologous *E. coli* host in a manner like those previously described^4,23^. Briefly, a single colony of R995 + PduST^23^ was used to inoculate 200 mL of LB supplemented with 50 µg/mL kanamycin and 0.5% (v/v) 1,2-propanediol and grown overnight at 37 °C in a baffled flask with shaking. Cells were harvested by centrifugation and resuspended in 40 mL of lysis buffer (20 mM Tris–HCl pH 8.0, 5 mM MgCl_2_, 200 mM KCl, 0.5 mM EDTA, 0.5 mM PMSF, 2 mM β-mercaptoethanol, and 0.5 mg/mL egg white lysozyme) and incubated at RT with mixing for 20 min. After, the lysate was cooled on ice and briefly sonicated for two seconds to clear excess gDNA. The lysate was then cleared at 12000xg for 10 min. Pdu BMCs were pelleted from the supernatant at 20000xg for 30 min and resuspended in 3 mL of cold assay buffer (20 mM potassium phosphate pH 8.0, 5 mM MgCl_2_, 50 mM KCl). Pdu BMCs were repelleted a second time at 20000xg for 20 min and resuspended in 1 mL of cold assay buffer and quantified via A_205_ on a Nanodrop One. Samples were stored at 4 °C until use later that same day. Denatured Pdu BMCs were produced by sonicating 1 mL of native BMCs in assay buffer on ice to prevent excess heating.

### Transmission electron microscopy

Native and broken Pdu BMCs were diluted to < 100 µg/mL into assay buffer before 5 µL was applied to a 400 mesh Lacey carbon grid (Ted Pella) for 15 min. Excess was wicked off and grids were desalted with three washes with deionized water. Samples were then negatively stained with UranyLess (EMS) stain for 1 min. Stained samples were imaged with a Thermo Talos L120C under TEM mode.

### Propanal evolution assay

Pdu BMCs (both native and broken) were diluted in assay buffer (20 mM potassium phosphate pH 8.0, 5 mM MgCl_2_, 50 mM KCl) supplemented with 0–500 mM of additional NaCl in 100 mM increments as appropriate and coenzyme B_12_ and incubated at RT for 10 min protected from light. After, 90 µL aliquots of each sample were distributed in quadruplicate in a UV-transparent 96-well plate. Reactions were initiated by addition of 10 µL of 2 M 1,2-propanediol diluted in appropriate buffer. The final concentrations of Pdu BMC, coenzyme B_12_, and 1,2-propanediol were 200 µg/mL, 15 µM, and 200 mM respectively. Samples were then placed into a pre-warmed (30 °C) Biotek plate reader with data collection initiated within 1 min of 1,2-propanediol addition. Absorbance at 280 nm was monitored in 30 s increments for 1 h. Data was analyzed in GraphPad Prism 9.

### MD simulations

Systems comprise one BMC shell protein PduA hexamer from *Salmonella enterica* (UniProt: P0A1C7) based on the conformation in PDB: 3NGK^16^. The 18 simulations (conducted under 6 different conditions) described in this manuscript are outlined in Table 1.

Residues missing in the PduA crystal structure (N-terminal M_1_QQ_3_ and C-terminal S_93_Q_94_) are omitted and N- and C-terminal backbones are capped with neutral NH_2_ and C(O)OH groups, respectively. Histidine residues are neutral and protonated on N_ε_. S40A mutations are enacted by removing all S40 O_γ_ atoms. Crystal waters are retained. A cubic unit cell is constructed with a 4 nm minimum distance between periodic protein images (~ 11.5 nm box edge length). For systems containing metabolites, an initial conformation of S-1,2-propanediol (SMILES: OCC(O)C) or propanal (SMILES: CCC=O) is obtained with RdKit^41^ and added at 175 mM with random rotations and translations. Each system is hydrated with ~ 47,000 water molecules. For systems containing salt, 150 mM NaCl is added by replacing randomly selected non-crystallographic water molecules at least 1 nm away from any protein or crystallographic water atom. Each of the 18 systems is independently constructed and equilibrated.

All simulations are run with GROMACS 2022.2^42^. Protein is modeled by the CHARMM36m force field with CMAP corrections^43^. Parameters for 1,2-propanediol and propionaldehyde are obtained from CGenFF^44^ and converted to GROMACS format with the cgenff_charmm2gmx_py3_nx2.py script (downloaded from https://mackerell.umaryland.edu/charmm_ff.shtml on September 15, 2022). The water model is TIP3P^45^ with CHARMM modifications^46^. Water molecules are rigidified with SETTLE^47^ and other covalent bond lengths involving hydrogen are constrained with P-LINCS^48^ (maximum order of 6). Lennard–Jones (LJ) interactions are evaluated using an atom-based cutoff with forces switched smoothly to zero between 1.0 and 1.2 nm. Coulomb interactions are calculated using the smooth particle-mesh Ewald method^49,50^ with Fourier grid spacing of 0.12 nm and fourth order interpolation. Dual 8 × 8 non-bonded neighbor-lists are dynamically updated to 1.205 and 1.346 nm every 12 and 100 integration steps, respectively, based on a Verlet buffer tolerance of 0.005 kJ/mol/ps. Initial configurations are subjected to 5,000 steps of steepest descent energy minimization in which non-hydrogen atoms in protein and crystal water molecules are fixed in place (excepting C-terminal protein backbone OT2 atoms, which are free to move in order to correct poor build geometries). Subsequent simulation in the isothermal-isobaric ensemble is achieved by isotropic coupling to the C-rescale barostat^51^ at 1.01325 bar with a compressibility of 4.5 × 10^−5^/bar and a coupling constant of 4 ps; temperature-coupling is achieved using the V-rescale thermostat﻿^52^ at 310 K with a single coupling group and a coupling constant of 1 ps. The integration time step is 2 fs. Following energy minimization, each system is simulated for 0.25 ns with the initial set of fixed atoms. Fixed atoms are then released and 1 ns of simulation is conducted with position restraints (force constant *F*_*c*_ = 1000 kJ/mol/nm^2^) on non-hydrogen protein atoms and crystal waters, followed by 1 ns only restraining protein backbone N, C_α_, and C atoms with *F*_*c*_ = 1000 kJ/mol/nm^2^ and another 1 ns with *F*_*c*_ = 100 kJ/mol/nm^2^, then 1 ns only restraining C_α_ atoms with *F*_*c*_ = 100 kJ/mol/nm^2^ and another 1 ns with *F*_*c*_ = 10 kJ/mol/nm^2^. All position restraints are then removed and production simulations are conducted for ~ 1.5 μs each (average duration = 1.49 μs, standard deviation = 0.08 μs).

Prior to analyses, each trajectory frame is aligned to minimize the sum squared displacement of protein hexamer C_α_ atoms with their initial crystallographic positions so as to align the PduA pore normal with the Cartesian z dimension. Quantifications of molecular displacement along this pore axis, *d*_*pore*_, use the center of geometry of each molecule of water, Cl^−^, Na^+^, 1,2-propanediol, and propionaldehyde. The average number each molecule along the pore axis, *N*_*mol*_, is evaluated within a cylinder of radius 0.7 nm whose central long axis lies along the pore normal (parallel to the Cartesian z dimension after realignment) and passes through the center of geometry of the C_α_ atoms of residue 40 in all six protein monomers (Fig. 3E). The center of geometry of these C_α_ atoms defines *d*_*pore*_ = 0 nm and the protein is oriented such that values of *d*_*pore*_ > 0 nm represent sampling along the hexamer’s concave face, which would be on the outside of an assembled BMC. To quantify permeation events in which a small molecule passes through the PduA pore (in either direction), we define a cylinder as outlined above but having a radius of 2 nm and extending only along − 0.6 nm ≤ *d*_*pore*_ ≤ 0.6 nm (Fig. 3A). A permeation event is counted whenever a molecule enters this cylinder one side of the pore and exits on the other side. The larger radius used to assess permeation (as compared to density evaluations) is intended to avoid spurious counts by ensuring that the permeant cannot exit and reenter the cylinder laterally near the axial center. Spatial distribution functions are computed with bin widths of 0.05 nm and local density ratio assessments use 0.09033 ions per nm as the expectation value for no enrichment given 150 mM Cl^−^.

### Supplementary Information

Supplementary Information 1.

Supplementary Video 1.

## Data Availability

The datasets generated and/or analyzed during the current study are available from the corresponding authors on reasonable request.
