# Supplementary material for: Monatomic ions influence substrate permeation across bacterial microcompartment shells
Source: Sci Rep. 2023 Sep 21;13:15738. doi: 10.1038/s41598-023-42688-9 (PMC10514305; doi:10.1038/s41598-023-42688-9)
Supplement: Supplementary file 1 — Supplementary Information 1. [file 41598_2023_42688_MOESM1_ESM.docx]

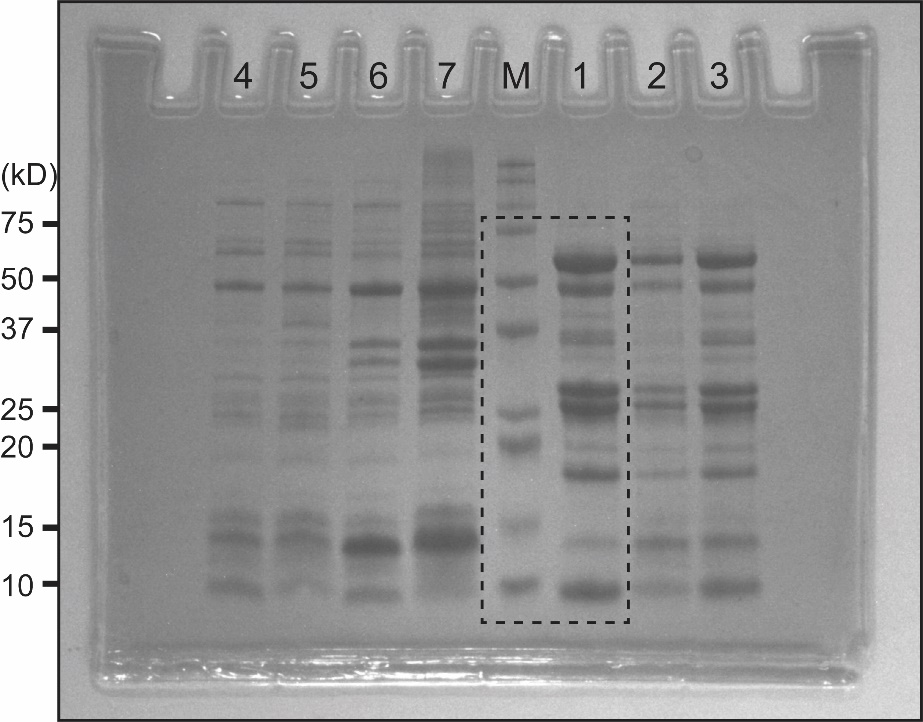
**Supplemental Information**

**Supplemental Figure 1: Uncropped SDS-PAGE of Pdu BMC purifications performed for this study, related to Figure 1c.** The inset denotes the gel section that was cropped for Figure 1c. Lanes 1-3 are replicates of Pdu BMC purifications. Lanes 4-7 are extraneous work unrelated to this study but ran in parallel. M, marker.


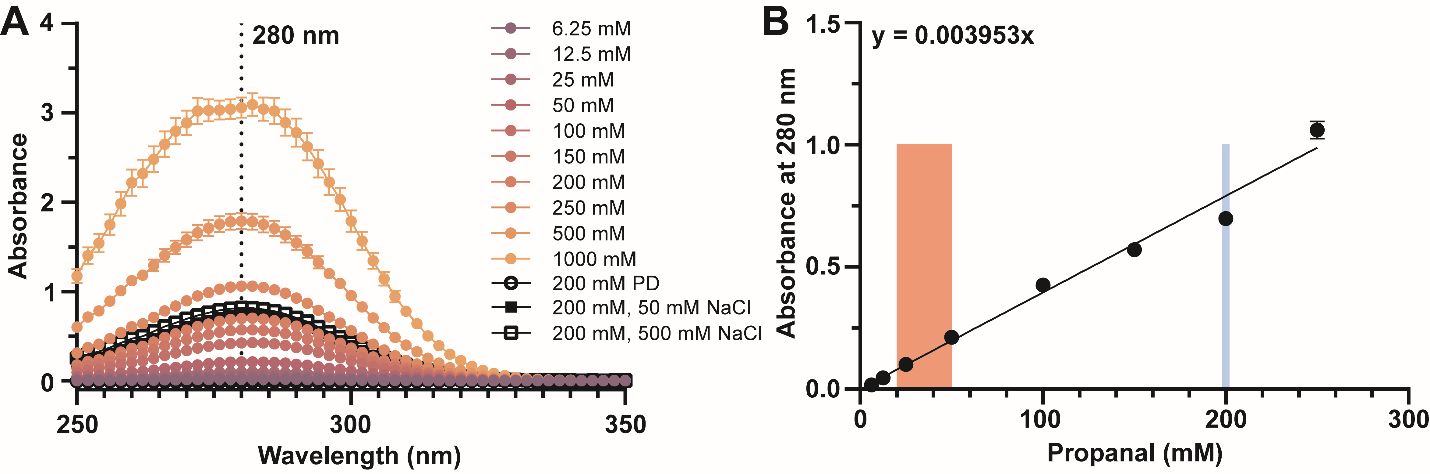


**Supplemental Figure 2: Direct spectroscopic detection of propanal.** (A) Solutions of propanal in assay buffer were prepared from 0-1000 mM and the absorbance was measured from 250-350 nm. Propanal had a peak absorption at 280 nm. 1,2-propanediol (PD) could not be detected. Propanal absorption was not significantly affected by additional NaCl. All measurements were performed in triplicate. (B) Propanal detection was linear up to 250 mM. A linear regression was performed to aid in quantification. The blue shaded region represents the concentration of 1,2-propanediol used in activity assays (200 mM) and the orange shaded region represents the range of propanal output (20-50 mM) within the linear region.


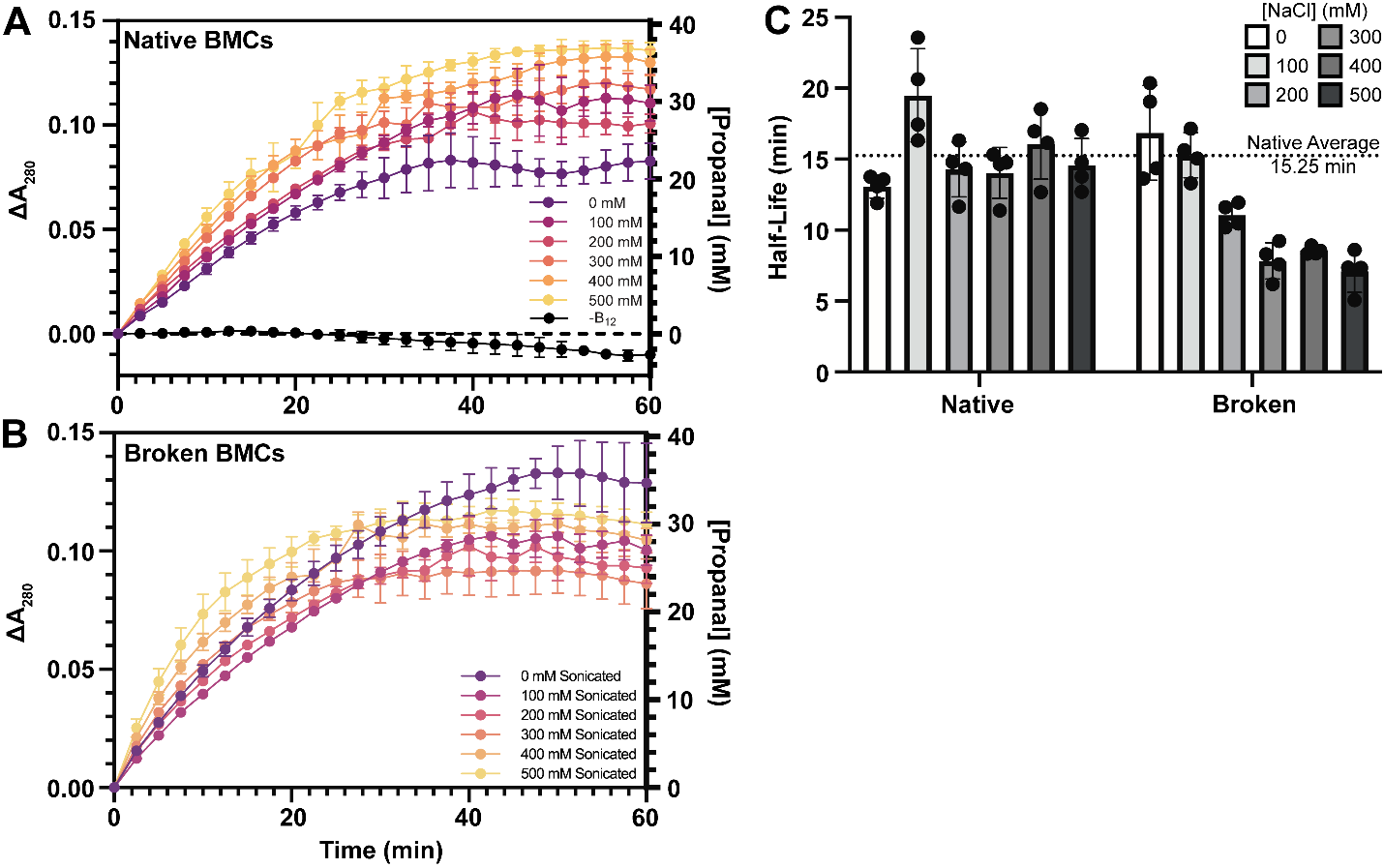


**Supplemental Figure 4: Average RMSD of each residue across six monomers of pduA with different conditions.** The inset shows a zoomed-in view of the GSG/GAG motif. The error bars represent standard deviations of average values calculated from three independent runs. Note that three lines with higher peaks at G39 are blue, orange, and purple.

**Supplemental Figure 3: Full propanal evolution kinetic curves.** (A) The kinetic profiles of native BMCs in different [NaCl]. (B) The kinetic profiles of BMCs broken by sonication in different [NaCl]. The reactions performed in (A) and (B) were performed in quadruplicate. (C) The half-lives of the reaction profiles from (A) and (B). The half-lives were determined by fitting individual curves to the one-phase exponential association equation in GraphPad Prism 9. The rate constant, K, was used to determine the half-lives which were then averaged for each condition.

**Supplemental Figure 5: (A) Top view and (B) side view of groups used to calculate bending angles in the pduA hexamer.** Two types of bending angles are calculated, i.e., Ax-center-Ay and Bx-center-By, where x and y represent two opposed monomers. The “center” group is alpha-C atoms of residue 39-41 for every monomer. The “Ax” groups are alpha-C atoms of residue 53-63 for every monomer. The “Bx” groups are alpha-C atoms of residue 31-35 and residue 45-49 for every monomer. The center-of-mass (COM) for each group is used for the calculation of bending angles.

**Supplemental Figure 6: Time evolutions of AX-center-AY angles and BX-center-BY angles for pduA hexamers with 1,2-propanediol (left) with salt and (right) without salt.** The average values and standard deviations of bending angles are shown in Table R1.

**Supplemental Table 1: Ax-center-Ay and Bx-center-By bending angles. The uncertainties are calculated from standard deviations of average values for Ax-center-Ay and Bx-center-By angle.7**

| **Bending angle (degree)** | **With Salt** | **Without Salt** |
| --- | --- | --- |
| **A0-center-A3** | 176.29 | 173.61 |
| **A1-center-A4** | 176.21 | 173.79 |
| **A2-center-A5** | 175.68 | 176.30 |
| **Ax-center-Ay** | 176.06 $\pm$ 0.27 | 174.57 $\pm$ 1.23 |
| **B0-center-B3** | 136.04 | 129.55 |
| **B1-center-B4** | 135.08 | 131.67 |
| **B2-center-B5** | 131.32 | 135.39 |
| **Bx-center-By** | 134.15 $\pm$ 2.04 | 132.20 $\pm$ 2.41 |

**
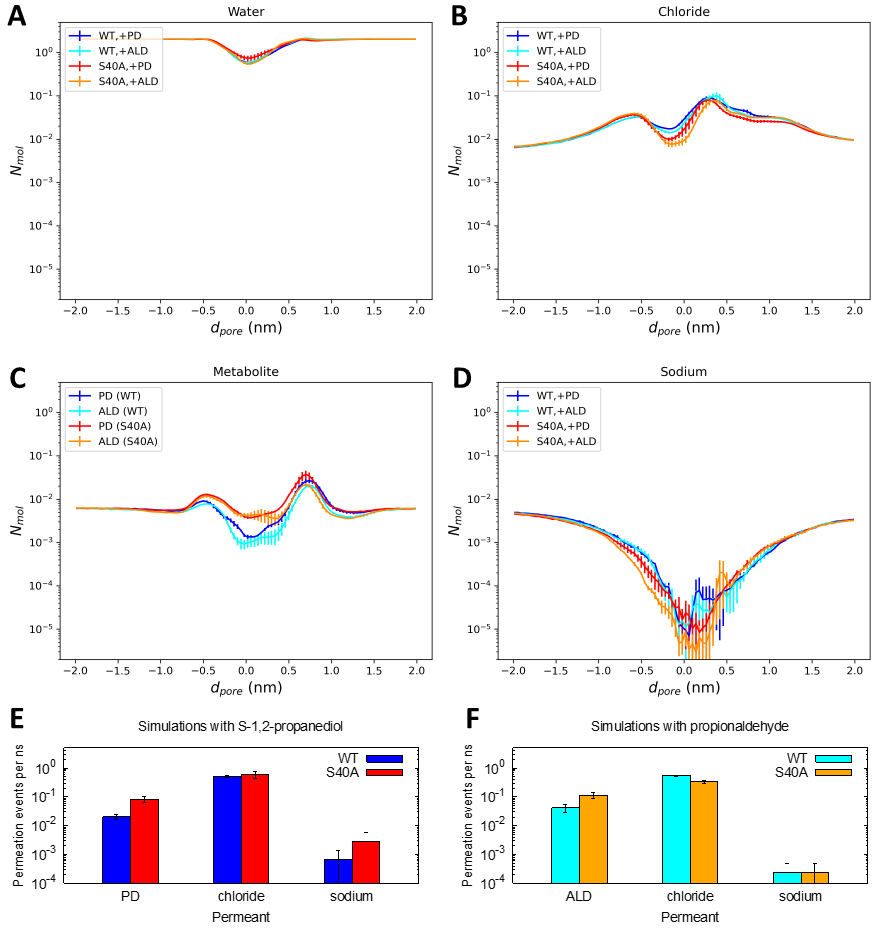
**

**Supplemental Figure 7: Molecular occupancy and permeation of 1,2-propanediol and propionaldehyde through WT and S40A PduA in MD simulations.** (A-D) Histograms of the average number each molecule, Nmol, at various displacements along a 0.7 nm-radius cylinder aligned with the PduA pore axis, dpore, referenced such that the average position of PduA C_α_ atoms in residue 40 is at dpore = 0 nm. Data shown separately for (A) water, (B) Cl^-^, (C) metabolite, and (D) Na^+^. Metabolite is either 1,2-propanediol (PD) or propionaldehyde (ALD). Protomers are either WT or the S40A variant. All simulations depicted in this figure have 150 mM NaCl and 175 mM metabolite. Vertical bars represent standard errors across three repeat simulations. (E and F) Rates of bidirectional molecular permeation through the PduA pore. Permeants are labeled on the abscissa and the metabolite is either (E) PD (1,2-propanediol) or (F) ALD (propanal).

**Supplemental Movie 1: Simulation of 1,2-propanediol permeation through the PduA pore.** The movie is a 1 ns snippet from the 1.5 µs simulations used for data collection. 1,2-propanediol is shown in stick representation. The Cl^-^ and Na^+^ ions are shown as yellow and blue spheres, respectively. Water molecules are omitted for clarity.
